# Impact of High-Grade Glioma Lesion Location on Preoperative Neuropsychological Deficits

**DOI:** 10.3390/cancers17172775

**Published:** 2025-08-26

**Authors:** Ethan J. Houskamp, Emmalee L. Skorich, Melissa-Ann Mackie, Matthew C. Tate

**Affiliations:** 1 Northwestern University Feinberg School of Medicine, Chicago, IL 60611, USA; ethan.houskamp@northwestern.edu; 2 Department of Medicine, Northwestern University Feinberg School of Medicine, Chicago, IL 60611, USA; emmalee.skorich@gmail.com; 3 Department of Psychiatry and Behavioral Sciences, Northwestern University Feinberg School of Medicine, Chicago, IL 60611, USA; melissa-ann.mackie@nm.org; 4 Department of Neurological Surgery, Northwestern University Feinberg School of Medicine, Chicago, IL 60611, USA; 5 Department of Neurology, Northwestern University Feinberg School of Medicine, Chicago, IL 60611, USA

**Keywords:** neuro-oncology, brain mapping, neuroimaging

## Abstract

**Background**: Glioblastoma (GBM) is an aggressive brain tumor, with surgery being an integral part of treatment. Aggressive resections improve clinical outcomes but need to be balanced against potential functional impairment. Neuropsychological (NP) testing is an important tool neurosurgeons use to assess cognitive functioning. Importantly, associations between NP test scores and imaging biomarkers could enable a testable baseline by which to track patient outcomes over time and aid in presurgical counseling. **Methods**: We identified 44 patients diagnosed with primary GBM and who had detailed NP testing and presurgical imaging. Regression models for NP indices were created with tumor size, hemisphere, and lobar location as predictors. Lesion–symptom mapping (LSM) analyses were used to identify more detailed structure–function relationships. **Results**: Larger tumor volumes predicted worse attention, immediate memory, language, visuospatial, and overall NP performance (*p* < 0.05 for all). Left hemisphere involvement predicted worse attention, language, and immediate memory NP performance (*p* < 0.01 for all). Only visuospatial testing had lobar location significantly associated with worse scores (occipital lobe; *p* < 0.05). The LSM analyses identified areas around the left sagittal stratum as significantly associated with language performance (*p* < 0.05), with no other structure–function relationships being identified. **Conclusions**: These findings support the growing evidence that outside of a small number of truly critical regions, high-grade gliomas impair cognition generally, likely due to progressive tumor infiltration-associated neuroplasticity of complex parallel and interconnected networks. To investigate this, future studies should incorporate larger cohort sizes and should examine the relationship of glioma-induced network-level perturbations on cognitive decline.

## Simple Summary

Patients with brain tumors face an intrinsic tension while undergoing surgical resection. More extensive resections promote longer survival but also increase the risk of decline in cognition and quality of life. Neuropsychological tests are one tool used to inform clinical decision making before and after surgery. The aim of our retrospective study was to evaluate relationships between presurgical imaging biomarkers and neuropsychological scores in patients with newly diagnosed glioblastoma. If identified, such relationships may help guide clinical decision making. The cohort (n = 44) scored worse on the cognitive tests compared to matched cohorts. Tumor volume and left hemisphere involvement were associated with worse scores across multiple test cognitive functions. Only language function localized on lesion-based analysis, implicating primarily the left sagittal stratum. The paucity of structure–function relationships identified highlights the importance of neuroplasticity, even in the high-grade glioma population.

## 1. Introduction

Glioblastoma (GBM) is an aggressive malignant brain tumor originating from the brain’s glial cells. It is the most common primary brain tumor, with incidence being reported as high as 4.17 per 100,000 person-years [1]. There is currently no cure for GBM, and due to its aggressive nature, maximal safe resection followed by chemotherapy and radiation constitutes standard treatment. While increasing the extent of resection, including supratotal resection, is associated with improved survival outcomes, this must be balanced against the potential for causing new functional impairments [2,3,4,5]. To identify a resection strategy that maximizes onco-functional balance, it is important to understand the relationship between regions critical for functioning and overt tumor location. In this context, functional MRI (fMRI; both task-based and resting state), diffusion tensor imaging (DTI), magnetoencephalography (MEG), and navigated transcranial magnetic stimulation (nTMS) can each be used individually or in concert to aid in identifying important functional brain regions [6,7,8,9]. Candidate functional areas identified by preoperative mapping studies and/or functions determined to be at risk based on preoperative neuropsychological testing can then be refined intraoperatively via gold standard direct electrical cortical/subcortical stimulation-based (DES) mapping to identify truly critical brain regions. Thus, DES assists the neurosurgeon in resecting as much tumor-involved brain as possible while avoiding injury to key functional areas which, if removed, would cause significant functional impairment [5].

A key component of maximizing onco-functional balance in glioma patients involves a detailed understanding of the patient’s neurocognitive functioning. For glioma patients, this regularly involves neuropsychological (NP) testing. Unlike other methods, NP testing assesses neurological deficits in more realistic frameworks and contexts, an important factor in determining quality of life [10]. Additionally, the information gained from the testing can be used to aid in realistic presurgical consultations, plans for postoperative rehabilitation, and management at tumor recurrence by tracking cognitive performance over time [10,11,12,13,14]. As such, research has historically focused on the relationships between the character of NP deficit and its interaction between tumor size, type, and quality of life as well as identifying and tracking the cognitive changes that occur throughout the GBM disease course, especially following tumor resection [15,16,17,18]. NP test scores have been associated with rather broad characteristics including high-grade glioma hemisphere and size [15,19,20]. However, even with large atlas-derived maps, there have still been challenges in robustly identifying relationships between more detailed glioma locations and preoperative NP test scores [21].

Because of the core role of imaging in the diagnosis and monitoring of patients with glioma, identifying associations between glioma location and preoperative NP test scores would be particularly helpful in identifying cognitive functions to track through a patient’s disease course, including intraoperatively. The present study investigates the relationship between cognitive performance and high-grade glioma imaging biomarkers via regression-based analyses and lesion–symptom mapping.

## 2. Materials and Methods

### 2.1. Patients

All patients presented to the authors’ institution with newly diagnosed and histologically confirmed glioblastoma. Patients were included if they completed presurgical NP testing and had available baseline brain MRI with a gadolinium-enhanced T1-weighted and T2-weighted fluid-attenuated inversion recovery (FLAIR) images. Patients were excluded if they had a previous history of glioma or radiation or a multifocal tumor. Tumor hemisphere and lobe location were collected along with patient demographics from the electronic health records. The protocol was approved by the Institutional Review Board IRB (STU00202399). Informed consent was obtained from patients upon beginning care.

### 2.2. Neuropsychological Testing

All patients underwent NP testing during presurgical planning. The Repeatable Battery for the Assessment of Neuropsychological Status (RBANS) assessment was selected for analysis given its standardization and ubiquity as a NP measure. All NP testing was completed by the same group of neuropsychologists. All NP test scores were normalized (i.e., z-transformed) to patient-specific cohorts based upon educational levels, age, and sex. Patients who exhibited a deficit (z ≤ −1.65; the lowest 5% of scorers in the matched cohort) were calculated. Three patients had incomplete RBANS indices information. To prevent biasing, the missing index values were imputed using Bayesian ridge regression.

### 2.3. MRI Data

The clinical MRI scans were performed using standard pre-surgical procedures. A Gd-enhanced T1 sequence (1 mm slice thickness) was used for contrast enhancement calculations and as a structural reference for normalization. Lesion masks of tumors were created in the native MRI space using a Gd-enhanced T1 sequence for each patient using MRIcron “https://www.nitrc.org/projects/mricron (accessed on 1 August 2023)”. Two-dimensional lesions masks were manually created in the transverse plane. A dual-reviewer system was initially used to generate 2D masks by E.J.H. and E.L.S. Upon confirmation of mask agreement, the remaining lesions were masked individually. Final 3D masks were generated by imputing and smoothing 2D lesions between slices. The lesion masks and scans were enantiomorphically segmented and registered to the Montreal Neurological Institute (MNI) space using SPM12 “https://www.fil.ion.ucl.ac.uk/spm/software/spm12 (accessed on 1 July 2024)” in MATLAB (Release 2023a, The MathWorks Inc., Natick, MA, USA).

### 2.4. Regression Analysis

To evaluate the impact of lesion lobe, hemisphere, and size on NP test scores, multivariate multiple linear regression models were run for individual RBANS and total RBANS scores. Lesion lobe involvement was encoded as a categorical variable, lesion hemisphere as a binary variable, and lesion volume as a continuous variable measured in cm^3^. The dependent variables were the z-scored RBANS indices. All regression statistics were run using Statsmodels.api in Python 3.12.

### 2.5. Lesion–Symptom Mapping

Voxel-based lesion–symptom mapping (VLSM) was used to identify more detailed associations between anatomic regions and worse NP performance. The MNI-registered lesion masks were used to calculate a voxel-based lesion–symptom mapping analysis. The voxels of each normalized T1-generated masks were analyzed for the presence of a lesion and correlated with NP test scores using NiiStat “https://www.nitrc.org/projects/niistat (accessed on 1 August 2024)” in MATLAB. Lesion masks were regressed on volume to control for the effect of tumor size on the RBANS index. A false discovery rate (FDR) was used to correct for the number of statistical tests, with significance being set at a corrected *p* value < 0.05. The minimum amount of overlap required for analysis was set to 6 participants.

Regions of interest (ROIs) composed of significant voxels from VLSM analysis were referenced to widely used atlases to calculate anatomy statistics. The Harvard–Oxford atlas was used to evaluate ROI involvement of cortical and subcortical regions [22]. The Johns Hopkins DTI-based atlas was used to evaluate ROI involvement of the white matter tracts [23]. To prevent biasing of atlas locations due to different sizes, the 5 regions with the highest fraction (i.e., the proportion of an atlas region that overlaps the ROI produced by VLSM) and extension (i.e., the proportion of the ROI that overlaps with an atlas region) were calculated.

VLSM-based analyses with FDR corrections are conservative in their outcomes due to the correction accounting for the statistical tests run on each voxel. To achieve a description of lesion involvement that would be more likely to identify regions of statistical significance, we also conducted an atlas-based lesion mapping analysis using the same Harvard–Oxford and Johns Hopkins DTI-based atlases. In this analysis, regressions were performed on the involvement of lesion masks in the prespecified atlas regions rather than at the voxel level. Therefore, the atlas regions were specified prior to the symptom mapping and involvement of atlas regions formed the foundation of the regressions. The minimum overlap of the atlas-based analysis was set to 6 patients and determined by an adjusted *p* value less than 0.05.

## 3. Results

**Figure 1:**
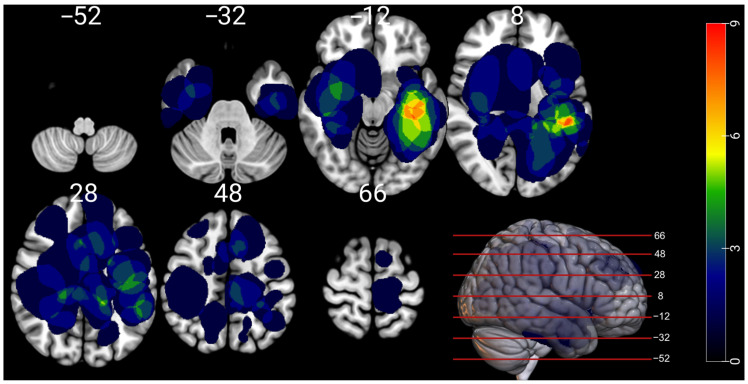
Overlap map of the 44 lesions normalized to MNI space. The scale indicates the number of lesions that overlap at a given voxel. The slice numbers indicate the z-coordinate in MNI space.

A total of 44 patients met the inclusion criteria. The cohort demographics are reported in Table 1. The most common lobes with lesions included the temporal (n = 19, 43.2%), frontal (n = 17, 38.6%), and parietal (n = 12, 27.3%) lobes. Figure 1 displays the overlap map of all the MNI-registered lesions. The mean tumor volume was 33.2 cm^3^ and the standard deviation was 31.5 cm^3^. The mean z-score of the total RBANS index was −0.92. Eleven (25.0%) patients scored in the fifth percentile or lower for the total RBANS score.

### 3.1. Regression

The RBANS attention index score worsened with increasing lesion volumes (estimate of effect = −0.015 per cm^3^, *p* = 0.009). Larger lesion volumes (estimate of effect = −0.027 per cm^3^, *p* < 0.001) and left hemisphere tumors (estimate of effect = −1.420 for left hemisphere, *p* = 0.006) were predictors of worse RBANS immediate memory index scores (Table 2). Larger lesion volumes (estimate of effect = −0.022 per cm^3^, *p* < 0.001) and left hemisphere tumors (estimate of effect = −1.235 for left hemisphere, *p* = 0.001) were also the only significant explanatory variables for the RBANS language index scores. Predictors of worse RBANS visuospatial index scores were increasing lesion volume (estimate of effect = 0.014 cm^3^, *p* = 0.036) and lesions involving the occipital lobe (estimate of effect = −1.297 for left hemisphere, *p* = 0.031). The only significant predictor of the total RBANS index score was lesion volume, with larger lesions being associated with worse total scores (estimate of effect = −0.022 cm^3^, *p* < 0.001). There were no significant explanatory variables for the RBANS delayed memory index score.

### 3.2. Lesion–Symptom Mapping

**Figure 2:**
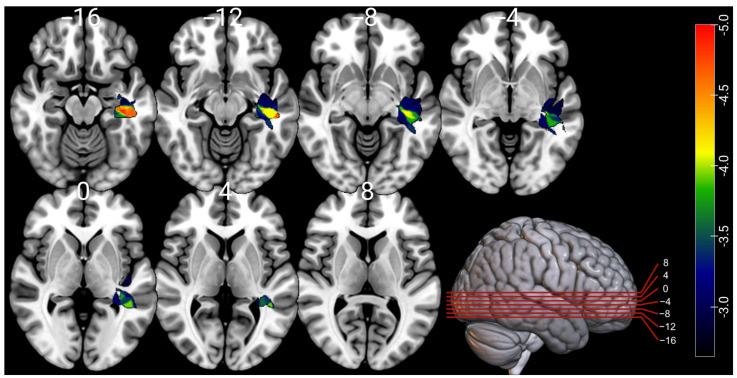
Voxel-based lesion–symptom mapping results of the RBANS language index. A total of 10,313 voxels survived threshold with significant z-scores < −2.67. The scale indicates the difference in normalized z-score results on the RBANS language index between those with lesions in the significant voxels and those with lesions not included in the group of significant voxels. Significance for results were corrected using FDR to an adjusted *p* value < 0.05.

Only the RBANS language task localized on the VLSM analysis (Figure 2). A total of 10,313 voxels survived the threshold with z-scores < −2.67 to create a language-derived region of interest (ROI). The cortical and subcortical areas with the highest fraction of overlap with the language-derived ROI were the left planum temporal (23.8%), posterior middle temporal gyrus (8.13%), posterior superior temporal gyrus (7.48%), posterior temporal fusiform cortex (7.34%), and posterior parahippocampal gyrus (5.44%). The cortical and subcortical areas with the highest extension into the ROI were the left planum temporal (10.0%), posterior middle temporal gyrus (9.6%), posterior superior temporal gyrus (6.7%), posterior temporal fusiform cortex (6.0%), and the insular cortex (3.8%). The white matter tracts with the highest fraction of overlap with the ROI were the left sagittal stratum (66.6%), retrolenticular part of the internal capsule (17.3%), stria terminalis (15.1%), posterior thalamic radiation (6.6%), and external capsule (2.3%). The white matter tracts with the greatest amount of extension into the ROI were the left sagittal stratum (21.2%), retrolenticular part of the internal capsule (5.4%), posterior thalamic radiation (3.4%), stria terminalis (2.8%), and external capsule (1.8%).

RBANS language tasks remained the only index to identify regions identified as significant in the atlas-based lesion analysis. All cortical and subcortical ROIs implicated at a *p* value < 0.05 in the analysis were within the left temporal lobe, including the left anterior superior temporal gyrus (STG, z = −4.19), left posterior inferior temporal gyrus (ITG, z = −4.31), left temporooccipital ITG (z = −5.13), left temporal fusiform cortex (TFC, z = −5.32), and left planum temporal (z = −3.32). The white matter tracts implicated at a *p* value < 0.05 also localized to the left temporal lobe and included the left middle temporal gyrus left (z = −3.83), the left fusiform gyrus (z = −4.61), the left hippocampus (z = −3.77), the left sagittal stratum (z = −4.27), left temporal ventricle (z = −4.03), and the left posterior ITG (z = −5.38). Subcortical and cortical areas implicated included the left temporooccipital ITG (z = −3.49), the left temporal fusiform cortex (z = −4.54), the left temporal occipital fusiform cortex (z = −3.95), and the left hippocampus (z = −3.28).

## 4. Discussion

Patients that presented with newly diagnosed glioblastoma and completed preoperative NP testing had lower than average cognitive performance compared to age- and education-matched controls. Over 25% of patients scored in the fifth percentile or lower on the overall RBANS test. The poor overall performance on the RBANS test was most associated with larger tumor size and left hemisphere lesions. Upon regression, tumor size was also a significant predictor of worse RBANS indices and total RBANS scores except for delayed memory. Furthermore, left hemisphere lesions were also predictive of worse scores on the RBANS immediate memory and language indices. However, outside of hemispheric involvement, locational description at the lobar level showed little association with NP performance. Lesion-based analyses produced similar results. Only language testing showed localization of function on the VLSM- and atlas-based analyses. Analysis of language function identified a region within the left temporal lobe as particularly important for robust language function. These findings further support the profound impact that gliomas can have on general cognitive function without patients presenting with clear focal deficits.

The findings of this cohort were consistent with the previously reported literature. The preoperative impact of tumor volume and hemisphere involvement on NP tests has been widely reported and aligns with this cohort [15,19,20,24,25]. The cortical structure most implicated across both VLSM- and atlas-based analyses was the left planum temporale, an important structure involved in auditory linguistic processing [26,27]. The left sagittal stratum was the most implicated white matter structure involved. It contains multiple important white matter bundles, including middle/inferior longitudinal fasciculi, IFOF, and optic radiations, and thus is a critical functional component of networks connecting the temporal, parietal, occipital, and frontal lobes [28,29,30]. Of particular relevance to neurosurgery, direct electrical stimulation-elicited effects included visual disturbances, neglect, language disturbances, confusion and comprehension difficulties, and mentalizing disturbances, reinforcing its criticality in mediating normal brain function [31,32,33]. The findings of the importance of the sagittal stratum in our cohort suggests that the widespread functions enabled by the white matter structures of the sagittal stratum are not readily redistributed or compensated; thus, for tumors involving this region, particular care should be taken during presurgical counseling and if resection is undertaken that multimodal stimulation-based mapping is strongly considered.

The significant yet diffuse cognitive deficits that this cohort of patients with gliomas experienced adds to the growing body of evidence that gliomas’ impact on cognitive functioning is not as closely related to the brain region(s) of primary damage as once thought. This is reflected in the paucity of associations identified between tumor location and cognitive functions. The most probable reason for the lack of functional localization is glioma- related neuroplasticity. A glioma’s infiltrative growth enables its integration into and modification of circuits in ways that promote tumor growth and worsen cognition [34,35]. In a multivariate-based analysis and disconnection study investigating trends in NP outcomes post-resection in a cohort of 400 patients diagnosed with low-grade glioma, the authors noted minimal sustained focal functional impairments outside of a handful of structures composed primarily of white matter tracts [36]. The impact of neuroplasticity highlights the importance of parallel and interconnected networks. That is, except for a relatively small number of critical areas without redundancy or parallelization of connections, damage to many structures is often compensated for through significant neuroplasticity [37,38]. In this light, to account for neuroplasticity and the parallelization of function, the intactness of networks will likely better predict deficits caused by gliomas.

The present study has limitations that should be noted. One is the cohort size, as the smaller cohort limits the lesion overlap and statistical power. This is particularly important in combination with the additional limitation of selection bias, as patients only underwent NP testing if there was clinical suspicion for cognitive deficits significant enough to be factored into surgical decision making. For this reason, there was a relative paucity in lesion overlap outside of the left temporal lobe. This combination of issues limits the ability of VLSM to identify structure–function relationships outside of the temporal region and should be studied in larger cohorts. Additionally, the single-cohort design also lacks external validation. A larger sample size and multi-center cross-validation are also needed to support and generalize these findings. The RBANS-based NP testing, while standardized, was collected at a single time point and offers limited assessment of executive functioning, fluency, and working memory. Some patients received anti-convulsant for seizure treatment or prophylaxis or corticosteroid medications for glioma-related edema, which is known to impact cognitive functioning. Lastly, there is some degree of selection bias as patients undergoing NP testing were not chosen at random but rather were patients for whom the tumor was presumed to be near critical functional areas. Further research would benefit from looking at larger cohorts with pre- and post-surgical NP testing to identify the long-term cognitive trajectory of patients based upon cognitive deficit or location patterns.

## 5. Conclusions

These findings support the increasing body of evidence that high-grade gliomas, similarly to their low-grade counterparts, impair cognition broadly rather than producing specific deficits based upon location, likely due to progressive tumor infiltration of complex parallel and interconnected networks. Nonetheless, our data suggest that larger gliomas involving the left hemisphere and particularly those involving the temporal lobe cortex and the deep white matter tracts of the posterior left hemisphere such as the sagittal stratum should confer particular attention during surgical planning given high correlation with cognitive deficits. Future studies incorporating larger cohort sizes and examining the relationship of glioma-induced network-level perturbations on cognitive decline and plasticity are warranted.

## Figures and Tables

**Table 1:** Cohort demographics.

Demographics	n = 44
**Average Age (SD)**	61.3 (11.7)
**Female Sex (%)**	25 (56.8)
**Left Hemisphere Lesion (%)**	34 (77.3)
**Left Handedness (%)**	14 (31.2)
**Language Dominance (%)**	
*Right*	30 (68.2)
*Unknown*	13 (29.5)
*Bilateral*	1 (2.3)
*Left*	0 (0.0)
**Lobe Involvement (%)**	
*Temporal*	19 (43.2)
*Frontal*	17 (38.6)
*Parietal*	12 (27.3)
*Occipital*	5 (11.4)
*Insula*	4 (9.1)
**Mean Tumor Size, cm^3^ (SD)**	33.2 (31.5)
**RBANS Total Score (SD)**	−0.92 (1.22)
*Lowest 5% of Scores (%)*	11 (25.0)

**Table 2:** Multivariate linear regression of RBANS indices by lesion lobe, hemisphere, and volume (cm^3^).

Attention Index	Estimate	Std. Error	*p* Value	95% Confidence Interval
**Constant**	0.058	0.605	0.924	[−1.167, 1.283]
**Frontal Lobe**	−0.456	0.359	0.212	[−1.184, 0.271]
**Parietal Lobe**	−0.042	0.35	0.905	[−0.751, 0.667]
**Occipital Lobe**	−0.674	0.502	0.188	[−1.692, 0.344]
**Temporal Lobe**	−0.13	0.348	0.711	[−0.836, 0.576]
**Lesion Hemisphere**	0.118	0.404	0.773	[−0.701, 0.936]
**Lesion Volume (cm^3^)**	−0.015	0.006	**0.009**	[−0.027, −0.004]
**Delayed Memory Index**	**Estimate**	**Std. Error**	***p* Value**	**95% Confidence Interval**
**Constant**	−0.332	0.843	0.696	[−2.040, 1.376]
**Frontal Lobe**	0.583	0.5	0.252	[−0.432, 1.597]
**Parietal Lobe**	0.287	0.488	0.56	[−0.701, 1.275]
**Occipital Lobe**	−0.311	0.7	0.66	[−1.730, 1.108]
**Temporal Lobe**	0.12	0.486	0.806	[−0.864, 1.104]
**Lesion Hemisphere**	−0.361	0.563	0.525	[−1.502, 0.780]
**Lesion Volume (cm^3^)**	−0.014	0.008	0.075	[−0.030, 0.002]
**Immediate Memory Index**	**Estimate**	**Std. Error**	***p* Value**	**95% Confidence Interval**
**Constant**	1.212	0.726	0.103	[−0.259, 2.682]
**Frontal Lobe**	−0.027	0.431	0.95	[−0.900, 0.846]
**Parietal Lobe**	−0.3	0.42	0.479	[−1.151, 0.550]
**Occipital Lobe**	−0.259	0.603	0.671	[−1.480, 0.963]
**Temporal Lobe**	−0.088	0.418	0.835	[−0.935, 0.760]
**Lesion Hemisphere**	−1.42	0.485	**0.006**	[−2.403, −0.438]
**Lesion Volume (cm^3^)**	−0.027	0.007	**<0.001**	[−0.040, −0.013]
**Language Index**	**Estimate**	**Std. Error**	***p* Value**	**95% Confidence Interval**
**Constant**	1.018	0.532	0.063	[−0.060, 2.095]
**Frontal Lobe**	0.315	0.316	0.324	[−0.324, 0.955]
**Parietal Lobe**	0.305	0.308	0.328	[−0.318, 0.928]
**Occipital Lobe**	−0.008	0.442	0.985	[−0.904, 0.887]
**Temporal Lobe**	−0.204	0.306	0.51	[−0.825, 0.417]
**Lesion Hemisphere**	−1.235	0.355	**0.001**	[−1.955, −0.515]
**Lesion Volume (cm^3^)**	−0.022	0.005	**<0.001**	[−0.032, −0.012]
**Visuospatial Index**	**Estimate**	**Std. Error**	***p* Value**	**95% Confidence Interval**
**Constant**	−0.737	0.696	0.296	[−2.148, 0.673]
**Frontal Lobe**	−0.161	0.413	0.698	[−0.999, 0.676]
**Parietal Lobe**	0.018	0.403	0.964	[−0.797, 0.834]
**Occipital Lobe**	−1.297	0.578	**0.031**	[−2.468, −0.125]
**Temporal Lobe**	0.687	0.401	0.095	[−0.125, 1.500]
**Lesion Hemisphere**	0.920	0.465	0.055	[−0.022, 1.862]
**Lesion Volume (cm^3^)**	−0.014	0.006	**0.036**	[−0.027, −0.001]
**Total Index**	**Estimate**	**Std. Error**	***p* Value**	**95% Confidence Interval**
**Constant**	0.263	0.615	0.671	[−0.983, 1.509]
**Frontal Lobe**	0.046	0.365	0.901	[−0.694, 0.785]
**Parietal Lobe**	0.047	0.356	0.895	[−0.674, 0.768]
**Occipital Lobe**	−0.658	0.511	0.206	[−1.693, 0.378]
**Temporal Lobe**	0.128	0.354	0.721	[−0.591, 0.846]
**Lesion Hemisphere**	−0.523	0.411	0.211	[−1.356, 0.309]
**Lesion Volume (cm^3^)**	−0.022	0.006	**<0.001**	[−0.033, −0.010]

## Data Availability

Data is contained within the Supplementary Material.

## Supplementary Materials

The following supporting information can be downloaded at: https://www.mdpi.com/article/10.3390/cancers17172775/s1. Supplemental data files.

## References

[1] Fabbro-Peray P., Zouaoui S., Darlix A., Fabbro M., Pallud J., Rigau V., Mathieu-Daude H., Bessaoud F., Bauchet F., Riondel A. (2019). Association of patterns of care, prognostic factors, and use of radiotherapy–temozolomide therapy with survival in patients with newly diagnosed glioblastoma: A French national population-based study. J. Neuro-Oncol..

[2] Ohka F., Natsume A., Wakabayashi T. (2012). Current trends in targeted therapies for glioblastoma multiforme. Neurol. Res. Int..

[3] Iacob G., Dinca E.B. (2009). Current data and strategy in glioblastoma multiforme. J. Med. Life.

[4] Scott J., Tsai Y.-Y., Chinnaiyan P., Yu H.-H.M. (2011). Effectiveness of radiotherapy for elderly patients with glioblastoma. Int. J. Radiat. Oncol. Biol. Phys..

[5] Mrugala M.M. (2013). Advances and challenges in the treatment of glioblastoma: A clinician’s perspective. Discov. Med..

[6] Shukla G., Alexander G.S., Bakas S., Nikam R., Talekar K., Palmer J.D., Shi W. (2017). Advanced magnetic resonance imaging in glioblastoma: A review. Chin. Clin. Oncol..

[7] Picht T., Frey D., Thieme S., Kliesch S., Vajkoczy P. (2016). Presurgical navigated TMS motor cortex mapping improves outcome in glioblastoma surgery: A controlled observational study. J. Neuro-Oncol..

[8] Abdullah K.G., Lubelski D., Nucifora P.G., Brem S. (2013). Use of diffusion tensor imaging in glioma resection. Neurosurg. Focus..

[9] Rossi M., Sciortino T., Conti Nibali M., Gay L., Viganò L., Puglisi G., Leonetti A., Howells H., Fornia L., Cerri G. (2021). Clinical Pearls and Methods for Intraoperative Motor Mapping. Neurosurgery.

[10] Ownsworth T., Hawkes A., Steginga S., Walker D., Shum D. (2009). A biopsychosocial perspective on adjustment and quality of life following brain tumor: A systematic evaluation of the literature. Disabil. Rehabil..

[11] Hales R.E. (2008). The American Psychiatric Publishing Textbook of Psychiatry.

[12] Armstrong C.L., Goldstein B., Shera D., Ledakis G.E., Tallent E.M. (2003). The predictive value of longitudinal neuropsychologic assessment in the early detection of brain tumor recurrence. Cancer.

[13] Meyers C.A., Hess K.R. (2003). Multifaceted end points in brain tumor clinical trials: Cognitive deterioration precedes MRI progression. Neuro Oncol..

[14] Meyers C.A., Brown P.D. (2006). Role and relevance of neurocognitive assessment in clinical trials of patients with CNS tumors. J. Clin. Oncol..

[15] Talacchi A., Santini B., Savazzi S., Gerosa M. (2011). Cognitive effects of tumour and surgical treatment in glioma patients. J. Neuro-Oncol..

[16] Rijnen S.J.M., Butterbrod E., Rutten G.M., Sitskoorn M.M., Gehring K. (2020). Presurgical Identification of Patients With Glioblastoma at Risk for Cognitive Impairment at 3-Month Follow-up. Neurosurgery.

[17] Tucha O., Smely C., Preier M., Lange K.W. (2000). Cognitive deficits before treatment among patients with brain tumors. Neurosurgery.

[18] Butterbrod E., Bruijn J., Braaksma M.M., Rutten G.M., Tijssen C.C., Hanse M.C.J., Sitskoorn M.M., Gehring K. (2019). Predicting disease progression in high-grade glioma with neuropsychological parameters: The value of personalized longitudinal assessment. J. Neuro-Oncol..

[19] Santini B., Talacchi A., Squintani G., Casagrande F., Capasso R., Miceli G. (2012). Cognitive outcome after awake surgery for tumors in language areas. J. Neuro-Oncol..

[20] Satoer D., Visch-Brink E., Smits M., Kloet A., Looman C., Dirven C., Vincent A. (2014). Long-term evaluation of cognition after glioma surgery in eloquent areas. J. Neuro-Oncol..

[21] Boelders S.M., De Baene W., Postma E., Gehring K., Ong L.L. (2024). Predicting Cognitive Functioning for Patients with a High-Grade Glioma: Evaluating Different Representations of Tumor Location in a Common Space. Neuroinformatics.

[22] Desikan R.S., Ségonne F., Fischl B., Quinn B.T., Dickerson B.C., Blacker D., Buckner R.L., Dale A.M., Maguire R.P., Hyman B.T. (2006). An automated labeling system for subdividing the human cerebral cortex on MRI scans into gyral based regions of interest. Neuroimage.

[23] Hua K., Zhang J., Wakana S., Jiang H., Li X., Reich D.S., Calabresi P.A., Pekar J.J., van Zijl P.C., Mori S. (2008). Tract probability maps in stereotaxic spaces: Analyses of white matter anatomy and tract-specific quantification. Neuroimage.

[24] Chang W.H., Wei K.C., Chen P.Y., Chen Y.C., Wu Y.Y., Tsai H.C., Chen M.H., Chao Y.P., Chen K.T. (2023). The impact of patient factors and tumor characteristics on language neuroplasticity in left hemispheric diffuse gliomas prior to surgical resection. J. Neuro-Oncol..

[25] Dallabona M., Sarubbo S., Merler S., Corsini F., Pulcrano G., Rozzanigo U., Barbareschi M., Chioffi F. (2017). Impact of mass effect, tumor location, age, and surgery on the cognitive outcome of patients with high-grade gliomas: A longitudinal study. Neuro-Oncol. Pract..

[26] Binder J.R., Frost J.A., Hammeke T.A., Rao S.M., Cox R.W. (1996). Function of the left planum temporale in auditory and linguistic processing. Brain.

[27] Shapleske J., Rossell S.L., Woodruff P.W.R., David A.S. (1999). The planum temporale: A systematic, quantitative review of its structural, functional and clinical significance. Brain Res. Rev..

[28] Maldonado I.L., Destrieux C., Ribas E.C., Siqueira de Abreu Brito Guimarães B., Cruz P.P., Duffau H. (2021). Composition and organization of the sagittal stratum in the human brain: A fiber dissection study. J. Neurosurg..

[29] Latini F., Trevisi G., Fahlström M., Jemstedt M., Alberius Munkhammar Å., Zetterling M., Hesselager G., Ryttlefors M. (2021). New Insights Into the Anatomy, Connectivity and Clinical Implications of the Middle Longitudinal Fasciculus. Front. Neuroanat..

[30] Gülsuna B., Güngör A., Yazgan P., Erol G., Middlebrooks E.H., Börcek A.Ö., Weninger W.J., Türe U. (2024). Revisiting the microsurgical anatomy of the sagittal stratum and surgical implications: Fiber microdissection and tractography study. J. Neurosurg..

[31] Duffau H. (2008). The anatomo-functional connectivity of language revisited. New insights provided by electrostimulation and tractography. Neuropsychologia.

[32] Chan-Seng E., Moritz-Gasser S., Duffau H. (2014). Awake mapping for low-grade gliomas involving the left sagittal stratum: Anatomofunctional and surgical considerations: Clinical article. J. Neurosurg..

[33] Berro D.H., Herbet G., Duffau H. (2021). New insights into the anatomo-functional architecture of the right sagittal stratum and its surrounding pathways: An axonal electrostimulation mapping study. Brain Struct. Funct..

[34] Venkatesh H.S., Morishita W., Geraghty A.C., Silverbush D., Gillespie S.M., Arzt M., Tam L.T., Espenel C., Ponnuswami A., Ni L. (2019). Electrical and synaptic integration of glioma into neural circuits. Nature.

[35] Krishna S., Choudhury A., Keough M.B., Seo K., Ni L., Kakaizada S., Lee A., Aabedi A., Popova G., Lipkin B. (2023). Glioblastoma remodelling of human neural circuits decreases survival. Nature.

[36] Ng S., Moritz-Gasser S., Lemaitre A.-L., Duffau H., Herbet G. (2024). Multivariate mapping of low-resilient neurocognitive systems within and around low-grade gliomas. Brain.

[37] Duffau H. (2005). Lessons from brain mapping in surgery for low-grade glioma: Insights into associations between tumour and brain plasticity. Lancet Neurol..

[38] Duffau H. (2014). The huge plastic potential of adult brain and the role of connectomics: New insights provided by serial mappings in glioma surgery. Cortex.

